# Socio-economic status and behavioural and cardiovascular risk factors in Papua New Guinea: A cross-sectional survey

**DOI:** 10.1371/journal.pone.0211068

**Published:** 2019-01-23

**Authors:** Patricia Rarau, Justin Pulford, Hebe Gouda, Suparat Phuanukoonon, Chris Bullen, Robert Scragg, Bang Nguyen Pham, Barbara McPake, Brian Oldenburg

**Affiliations:** 1 Papua New Guinea Institute of Medical Research, Goroka, Eastern Highlands Province, Papua New Guinea; 2 School of Population Health, The University of Auckland, Auckland, New Zealand; 3 Nossal Institute for Global Health, University of Melbourne, Melbourne, Victoria, Australia; 4 Melbourne School of Population and Global Health, University of Melbourne, Melbourne, Victoria, Australia; Dasman Diabetes Institute, KUWAIT

## Abstract

**Background:**

Risk factors for cardiovascular disease (CVD) are negatively correlated with socio-economic status (SES) in high-income countries (HIC) but there has been little research on their distribution by household SES within low-and middle-income countries (LMICs). Considering the limited data from LMICs, this paper examines the association between behavioural and cardiovascular risk factors and household SES in Papua New Guinea (PNG).

**Methods:**

Reported here are results of 671 participants from the 900 randomly selected adults aged 15–65 years. These adults were recruited from three socioeconomically and geographically diverse surveillance sites (peri-urban community, rural Highland and an Island community) in PNG in 2013–2014. We measured their CVD risk factors (behavioural and metabolic) using a modified WHO STEPS risk factor survey and analysis of blood samples. We assessed SES by education, occupation and creating a household wealth index based on household assets. We calculated risk ratios (RR) and their 95% confidence intervals (CI) using a generalized linear model to assess the associations between risks and SES.

**Findings:**

Elevated CVD risk factors were common in all SES groups but the CVD metabolic risk factors were most prevalent among homemakers, peri-urban and rural highlands, and the highest (4^th^ and 5^th^) wealth quintile population. Adults in the highest wealth quintile had high risks of obesity, elevated HbA1c and metabolic syndrome (MetS) that were greater than those in the lowest quintile although those in the highest wealth quintiles were less likely to smoke tobacco. Compared to people from the Island community, peri-urban residents had increased risks of increased waist circumference (WC) (RR: 1.67, 95%CI: 1.21–2.31), hypertension (RR: 2∙29, 95%CI: 1∙89–4.56), high cholesterol (RR: 2∙22, 95%CI: 1∙20–4∙10), high triglycerides (RR: 1∙49, 95%CI: 1∙17–1∙91), elevated HbA1c (RR: 5∙54, 95%CI: 1∙36–21∙56), and Metabolic syndrome (MetS) (RR: 2∙04, 95%CI: 1∙25–3∙32). Similarly, Rural Highland residents had increased risk of obesity (Waist Circumference RR: 1∙70, 95%CI: 1∙21–3∙38, Waist-Hip-Ratio RR:1∙48, 95%CI: 1∙28–1∙70), hypertension (RR: 2∙60, 95%CI: 1∙71–3∙95), high triglycerides (RR: 1∙34, 95%CI: 1∙06–1∙70) and MetS (RR: 1∙88, 95%CI: 1∙12–3∙16) compared to those in the rural Island site.

**Interpretation:**

CVD risk factors are common in PNG adults but their association with SES varies markedly and by location. Our findings show that all community members are at risk of CVD weather they are part of high or low SES groups. These results support the notion that the association between CVD risk factors and SES differ greatly accordingly to the type of SES measure used, risk factors and the population studied. In addition, our findings contribute further to the limited literature in LMIC. Longitudinal studies are needed to monitor changes in rapidly changing societies such as PNG to inform public health policy for control and prevention of NCDs in the country.

## Introduction

Cardiovascular diseases (CVDs) are leading causes of mortality throughout the world.[1–3] More than three quarters of all CVD-related deaths occur in low-and middle-income countries (LMICs), most commonly due to coronary heart disease or stroke.[1, 2]

The major risk factors for CVD are well described: tobacco smoking, high blood pressure, high total and low-density lipoprotein (LDL) cholesterol, type 2 diabetes mellitus (T2DM) and physical inactivity.[1, 3] The co-occurrence of cardiovascular risk factors and metabolic syndrome (MetS), a cluster of at least three of five CVD risk factors,[4] that is associated with the development of both CVDs and T2DM, further increases lifetime risk of a CVD event.[3, 5]

An inverse association between socio-economic status (SES) with CVDs and their risk factors has been reported in many high income countries (HIC).[6–8] A review of studies conducted in HICs reported higher risk of incidence of stroke among those with lower SES compared to those with higher SES.[8] Although complex, this association appears to be related to behavioural, environmental and early life factors that are more common among individuals of lower than higher SES, such as poorer access to health care, use of alcohol, tobacco and drugs, low birth weight and high rates of childhood illnesses.[9, 10]

The evidence for a similar association between CVD risk and SES in low- and middle-income countries (LMICs) is less clear. A recent review reported that high SES in LMICs was associated with a higher prevalence of CVD risk factors such as low physical activity and excessive consumption of salt and fat while low SES was associated with other forms of CVD risk such as tobacco and alcohol use and limited fruit and vegetable consumption.[11] Furthermore, these relationships varied for the different CVD risk factors.[11] An earlier review also reported variable prevalence of CVD risk factors among different socioeconomic groups in LMICs.[12] These variations have been attributed to the complex interplay between the underlying cultural, social, economic, environmental and political determinants of health [9, 13, 14], and more specifically, the stage and type of economic development [15] and the associated impact on dietary and behavioural patterns.[16]

The prevalence of CVD risk factors in the Western Pacific is substantial and growing.[17–19] Papua New Guinea (PNG) is no exception.[20, 21] A recent review article of general population prevalence studies indicated a substantial increase in CVD risk has occurred between 1960s and 2007 (submitted for publication). In addition, findings of a recent cross-sectional study showed a high prevalence of CVD risk factors among peri-urban residents compared to rural adults. [20] However, the pace and extent to which PNG populations are exposed to CVD risk factors differs greatly across ethnic and regional groups.[22]

Little is known about the distribution of CVD risk factors according to SES in PNG. Given the differences in sub-national economic development profiles, PNG offers a unique opportunity to study the relationship between SES and CVD risk in divergent economic contexts within a LMIC. Therefore, we aimed to measure the distribution of these risk factors in three sites that are at varying levels of socioeconomic development

## Materials and methods

### Study setting

PNG is a lower-middle income country of 7.9 million people. The country’s economy is heavily reliant on extractive industries, particularly the mining of minerals. More than 80% of the population still live in rural areas, the majority of whom are engaged in subsistence farming or cash cropping.[23] Populations within PNG differ by genetics, duration and exposure to globalization and development.[24–26]

Economic development (measured by fluctuations in GDP growth (annual %), which peaked at 18% in 1993 and again at 15% in 2014), [27] has led to rapid social and demographic changes that are unevenly distributed across the country, with provinces at various stages of development.[24, 28]

We collected data from people living in the three sites of the PNG Institute for Medical Research’s integrated Health and Demography Surveillance System (iHDSS): West Hiri (Central Province), Asaro (Eastern Highlands Province) and Karkar Island (Madang Province).The West Hiri (peri-urban) site comprises villages of Austronesian ancestry, distributed along the coastline north-west of and in close proximity to PNG’s capital city, Port Moresby.[29, 30] The villages with a population estimated at around 11531, surround a large gas processing plant and are located within an hour’s drive of Port Moresby, a rapidly growing city established over a century ago.[29] It is the most developed of the three sites, with good access to government services and infrastructure, such as education and health care, electricity and water supply. In contrast, the Asaro is a highlands rural site comprised of people who are of Non-Austronesian ancestry.[30]. The population live in hamlets around 40–45 kilometres north-east of the Eastern highlands town of Goroka. The Asaro people are primarily subsistence farmers, but many earn cash through smallholder production of coffee, employment on plantations and selling garden products.[29, 31] The people of Karkar Island are of both Austronesian and Non-Austronesian ancestry [32] living on a volcanic island 30 kilometres off the northern coastline of the provincial capital, Madang. Karkar is a rural site and the least developed site of the three; most adult residents are subsistence farmers or unskilled labourers and access to Madang is by boat to the mainland and then by road, a journey of at least an hour.[29]

### Study design

We conducted a cross-sectional, community-based survey on randomly selected adults in the three iHDSS sites between April 2013 and October 2014. Our methods have been published elsewhere [20] but in brief, the sampling frame was a full population census of the adult population of each site. Using a simple randomization procedure, 900 individuals were randomly selected to participate in the survey, of which we aimed to recruit 300 adult participants from each site, stratified by sex and three age groups (15–29, 30–44, 45–65 years). The sample size was calculated to confer a 80% power to detect 10% absolute difference in the proportion of most risk factors from all ages combined between sites.

### Study procedures

The eligibility criteria for study participants were: 15–65 years of age, residing in the iHDSS site and not pregnant (for females). Prior to participation, written informed consent was obtained from participants (18+ years) and the parents or guardians of those aged less than 18 years, We interviewed selected participants at their homes or community health facilities, using the modified WHO STEPS NCD Risk Factor Survey Questionnaire.[33] Domains included: demographics, self-reported health status, dietary/nutrition history, tobacco, betelnut and alcohol use, physical activity, participant history of NCD and/or associated treatments. The survey questionnaire was available in English and Tok Pisin (the language used by the study populations at all three sites) and was piloted extensively prior to survey commencement. All survey forms and procedures were completed at a single time point by a trained survey team. Furthermore, The NCD data were linked with household SES data collected in 2015, extracted from the iHDSS database.

### Measurement of cardiovascular risk factors

#### Metabolic risk factors

According to the International Diabetes Federation and WHO criteria, central obesity was defined as a waist circumference ≥94 cm (males) and ≥80cm (females) and/or by waist to hip ratio ≥0·90 and ≥0·85 for males and females, respectively.[4, 34] Hypertension was defined as the average of the three systolic and diastolic BP of ≥130/85 mmHg and/or those on treatment for hypertension.[4, 35] Non-fasting serum samples were analysed for lipids (cholesterol, triglyceride and HDL-Cholesterol) using Vitros 250/350 Biochemistry System from Ortho Clinical Diagnostics, in batches within a month of collection. Elevated total cholesterol (TC) and triglyceride (TG) levels were defined as levels of >6·2mmol/L and ≥2·3 mmol/L respectively. Low HDL-c levels was defined as <1mmol/L (males) and <1·3mmol/L (females).[4, 36] T2DM was diagnosed if the participant was on anti-diabetic drugs or if their haemoglobin A1c (HbA1c) was ≥6·5%.[4, 37]. We used the IDF definition for metabolic syndrome (MetS), characterised by having large waist circumference (males ≥94cm and females ≥80cm) as a marker of central obesity plus any two of the following: hypertension (≥130/85mmHg), HbA1c levels (≥6·5%), elevated triglycerides (≥1·7mmol/L) and low HDL-c (<1·0/mmol/L M & <1·3mmol/L F).[4]

#### Behavioural risk factors

We included behavioural risk factors associated with an increased risk of CVDs and diabetes.[38, 39] Definitions included: smoking, daily use of any form of tobacco product; alcohol use, any amount of alcohol consumption within the last 30 days; chewing betelnut (areca nut, betel leaf/bean and slaked lime), any amount of betelnut chewing within the last 30 days; low fruit and vegetable intake, consumption on five or fewer days per week; high sugar intake, adding at least 6 teaspoons of sugar to hot drink(s) daily or drinking at least 3 cans of soft drink per week; high fried food intake, consuming fried food on at least five days per week; and daily salt intake, adding salt and/or stock cubes directly to food each day.

### Socioeconomic factors

We used education, occupation and household wealth indices as markers for SES. Though interrelated, each of these indicators influences health status and outcome of individuals differently.[40] We therefore investigated the association between each individual SES indicator (i.e. education, occupation and wealth) and cardiovascular risk factors. Typically, household income comes from many sources that vary by season, making it difficult to collect income data. Therefore, we used a wealth index, an approach used by the World Bank and Demographic and Health Surveys (DHS) as a proxy measure of income/economic status.[41–43]

### Data analysis

We used STATA/SE version 14.0 for all data analyses. We constructed the household wealth index from household data including household durables or assets and characteristics of participants’ houses (type, floor materials used and electricity, number of bedrooms, toilet and water quality) and ownership of a bank account. Using principal component analysis (PCA) we constructed wealth quintiles, based on the following variables[41]: water quality (low, medium, high); toilet quality (low, medium, high); type of house (low, medium, high); material used for the floor (low, medium, high); electricity (yes/no); number of bedrooms in the house; ownership of a bank account (yes/no) and number of household assets owned. The list of household assets included: air conditioning; fan; tractor; truck; car; boat or canoe; fridge; freezer; generator; washing machine; computer; landline or mobile phone; cupboard; bedframes; watch/clock, radio; gas or electric cooker; table and chairs. For each site, household quintiles were generated using the “xtile” command which constructed a new variable with five categories; lowest, second, third, fourth and highest quintiles, representing poorest to richest groups, respectively.

We estimated cardiovascular risk factor prevalence with 95% CI and used Pearson’s chi-square and Fisher’s exact test, where appropriate, to assess inter-site and socioeconomic differences in the risk factors. Risk ratios (RR) were determined, using the log binomial regression and a modified Poisson regression with a robust error variance for the univariate analysis and multivariate analysis respectively. We used available case analysis to deal with missing values.[44]

### Ethics approval

The study was approved by the PNGIMR Institutional Review Board (IRB) and the PNG Medical Research Advisory Committee (MRAC) (IRB No. 1208, 23 March 2012; MRAC No 12.34, November 2012) and registered with the University of Melbourne, School of Population and Global Health Human Ethics Advisory Group (ID: 1647305.1). Written informed consent was obtained from individual participants (aged 18+ years) and from the parents or guardians of participants who were less than 18 years of age prior to study participation.

## Results

Table 1 presents the socio-economic and demographic characteristics of the study population (N = 671). The three populations were different in terms of education and employment, but comparable in terms of participants’ age and sex. A higher percentage (34∙8%) of the participants from West Hiri had at least a secondary level education, followed by Karkar participants (15∙1%) then Asaro (11%). Overall, 21% of the participants received some, or completed, secondary education and 4% had completed at least some tertiary education. A higher percentage of Asaro and Karkar Island participants were subsistence farmers (73% and 57% respectively) compared to West Hiri (8%). A higher percentage of participants from West Hiri are engaged in paid employment (34%) compared to Karkar Island and Asaro with only 5% in both sites.

**Table 1:** Sociodemographic characteristics of the study participants: Overall and by site. The values are numbers and percentages.

	Overall		West Hiri (peri-urban)		Karkar Island (rural Island)		Asaro (rural highlands)	
**Sex**	N = 671	**n (%)**	**N = 253**	**n (%)**	**N = 199**	**n (%)**	**N = 219**	**n (%)**
Male		308 (45∙90)		108 (42∙69)		92 (46∙23)		108 (49∙32)
Female		363 (54∙10)		145 (57∙31)		107 (53∙77)		111 (50∙68)
**Age-group (years)**	N = 671		N = 253		N = 199		N = 219	
15–29		187 (27∙87)		69 (27∙27)		62 (31∙16)		56 (25∙57)
30–44		225 (33∙53)		88 (34∙78)		58 (29∙15)		79 (36∙07)
45–65		259 (38∙60)		96 (37∙94)		79 (39∙7)		84 (38∙36)
**Education**	N = 671		N-253		N = 199		N = 219	
Primary or lower		449 (66∙92)		111 (43∙87)		162 (81∙41)		176 (80∙37)
Some/complete Secondary		142 (21∙16)		88 (34∙78)		30 (15∙08)		24 (10∙96)
Vocational/Tertiary		25 (3∙73)		20 (7∙91)		2 (1∙01)		3 (1∙37)
Don’t Know		55 (8∙20)		34 (13∙44)		5 (2∙51)		16 (7∙31)
**Employment**								
Unemployed/retired/student		100 (14∙90)		51 (20∙16)		32 (16∙08)		17 (7∙76)
Home duties		146 (21∙76)		88 (34∙78)		44 (22∙11)		14 (6∙39)
Subsistence/cash cropper		294 (43∙82)		21 (8∙30)		113 (56∙78)		160 (73∙06)
Paid employment		107 (15∙95)		86 (33∙99)		9 (4∙52)		12 (5∙48)
Not given		24 (3∙58)		7 (2∙77)		1 (0∙50)		16 (7∙31)
**Marital Status**								
Single/never married		136 (20∙27)		57 (22∙53)		48 (24∙12)		31 (14∙16)
Married		445 (66∙32)		159 (62∙85)		144 (72∙36)		142 (64∙84)
Separated/divorced		27 (4∙02)		6 (2∙37)		2 (1∙01)		19 (8∙68)
Widowed		27 (4∙02)		14 (5∙53)		3 (1∙51)		10 (4∙57)
Not given		36 (5∙37)		17 (6∙72)		2 (1∙01)		17 (7∙76)
	**N = 611**		**N = 230**		**N = 180**		**N = 201**	
**Wealth Index (Quintiles)**								
Lowest quintile		127 (20∙79)		46 (20)		36 (20)		45 (22∙39)
Second lowest quintile		118 (19∙31)		46 (20)		36 (20)		36 (17∙91)
Third quintile		122 (19∙97)		46 (20)		36 (20)		40 (19∙90)
Fourth quintile		122 (19∙97)		46 (20)		36 (20)		40 (19∙90)
Highest quintile		122 (19∙97)		46 (20)		36 (20)		40 (19∙90)

Table 2 shows the prevalence and RR of behavioral CVD risk factors by SES. Females were less likely to smoke tobacco (RR 0.34), less likely to consume alcohol (RR 0.2) and sugar (RR 0.68) than males. Most of the behavioral risk factors were highly prevalent among West Hiri and Asaro participants as compared to Karkar residents, except for tobacco smoking. West Hiri participants were more likely to consume alcohol (RR 6.84), more likely to have high sugar (RR 4.34), salt (RR 3.66) and fried food (RR 19.6) intake than Karkar Island participants, Positive associations were observed between adults with tertiary/vocational education and alcohol use (RR 2.41), less fruits and vegetables (RR 2.76), high salt (RR 1.45) and fried food intake (RR 1.52). Similarly, adults in paid employment were more likely to engage in most of the behavioral risk factors compared to home makers. Positive associations were observed between adults engaged in paid employment with tobacco smoking (RR 2.31), alcohol use (RR 3.49), intake of high salt (RR 1.22) and fried food (RR 1.34) than home makers. Adults in the highest quintile had relatively lower risks of tobacco smoking than those in the lowest wealth quintile group, however this was not significant.

**Table 2:** Univariate analysis of CVD behavioral risk factors by socioeconomic factors (sex, site, education, employment, wealth index).

Socioeconomic characteristics		Tobacco smoking! N = 204				Alcohol use ≠ N = 105				Betel nut chewing ¥ N = 335				Less fruit/vegetables intake # N = 163				High sugar intake € N = 171				Salt intake £ N = 455				Fried food intake β N = 441		
Sex	N	n (%)	RR	95% CI	N	n (%)	RR	95% CI	N	n (%)	RR	95% CI	N	n (%)	RR	95% CI	N	n (%)	RR	95% CI	N	n (%)	RR	95% CI	N	n (%)	RR	95% CI
Male	225	146 (64∙9)	1∙00		206	86 (41∙8)	1∙00		206	157 (76∙2)	1∙00		305	71 (23∙3)	1∙00		301	95 (31∙6)	1∙00		303	212 (70∙0)	1∙00		302	203 (67∙2)	1∙00	
Female	260	58 (22∙3)	0∙34	0∙27, 0∙44	231	19 (8∙23)	0∙20	0∙12, 0∙31	223	178 (79∙8)	1∙05	0∙95, 1∙16	363	92 (25∙4)	1∙09	0∙83, 1∙43	355	76 (21∙4)	0∙68	0∙52, 0∙88	360	243 (67∙5)	0∙96	0∙87, 1∙07	360	238 (66∙1)	0∙98	0∙88, 1∙10
		p<0∙001	p<0∙001			p<0∙001	p<0∙001			p-0∙37	p-0∙37			p-0∙54	p-0∙54			p-0∙003	p-0∙003			p-0∙50	p-0∙50			p-0∙76	p-0∙76	
**Site**																												
Karkar Island	157	78 (49∙7)	1∙00		152	10 (6∙6)	1∙00		131	113 (86∙3)	1∙00		199	1 (0∙5)	1∙00		192	18 (9∙4)	1∙00		196	46 (23∙5)	1∙00		197	3 (1∙5)	1∙00	
West Hiri	162	59 (36∙4)	0∙73	0∙57, 0∙95	140	63 (45∙0)	6∙84	3∙66, 12∙80	149	139 (93∙3)	1∙08	1∙0, 1∙17	252	148 (58∙7)	116∙87	16∙5, 827∙86	248	101 (40∙7)	4∙34	2∙73, 6∙91	250	215 (86∙0)	3∙66	2∙83, 4∙74	247	227 (91∙9)	60∙35	19∙62, 185∙61
			p-0∙018				p<0∙001				p-0∙057				p<0∙001				p<0∙001				p<0∙001				p<0∙001	
Asaro	166	67 (40∙4)	0∙81	0∙64, 1∙04	145	32 (22∙1)	3∙35	1∙71, 6∙57	149	83 (55∙7)	0∙65	0∙55, 0∙76	217	14 (6∙5)	12∙84	1∙70, 96∙74	216	52 (24∙1)	2∙57	1∙56, 4∙23	217	194 (89∙4)	8∙81	2∙95, 4∙93	218	211 (96∙8)	63∙56	20∙67, 195∙41
		p-0∙048	p-0∙094			p-0∙005	p<0∙001			p<0∙001	p<0∙001			p-0∙441	p-0∙013			p-0∙006	p<0∙001			p<0∙001	p<0∙001			p<0∙001	p<0∙001	
**Education**																												
Primary or lower	329	145 (44∙1)	1∙00		302	59 (19∙5)	1∙00		291	219 (75∙3)	1∙00		447	81 (18∙1)	1∙00		440	103 (23∙4)	1∙00		443	281 (63∙4)	1∙00		445	268 (60∙2)	1∙00	
Some/completed Secondary	94	38 (40∙4)	0∙92	0∙70, 1∙21	81	23 (28∙4)	1∙45	0∙96, 2∙20	78	68 (87∙2)	1∙16	1∙04, 1∙29	142	52 (36∙6)	2∙02	1∙51, 2∙71	139	46 (33∙1)	1∙41	1∙06, 1∙89	141	108 (76∙6)	1∙21	1∙08, 1∙36	140	106 (75∙7)	1∙26	1∙11, 1∙42
			p-0∙54				p-0∙08				p-0∙007				p<0∙001				p-0∙02				p-0∙001				p<0∙001	
Vocational/Tertiary	17	6 (35∙3)	0∙80	0∙42, 1∙54	17	8 (47∙1)	2∙41	1∙38, 4∙19	18	14 (77∙8)	1∙03	0∙80, 1∙33	24	12 (50∙0)	2∙76	1∙77, 4∙31	24	8 (33∙3)	1∙42	0∙79, 2∙57	24	22 (91∙7)	1∙45	1∙26, 1∙66	24	22 (91∙7)	1∙52	1∙32, 1∙75
		p-0∙68	p-0∙51			p-0∙012	p-0∙002			p-0∙07	p-0∙80			p<0∙001	p<0∙001			p-0∙05	p-0∙24			p<0∙001	p<0∙001			p<0∙001	p<0∙001	
**Employment**																												
Home duties	94	22 (23∙4)		1∙00	81	10 (12∙4)		1∙00	85	79 (92∙9)		1∙00	146	58 (39∙7)		1∙00	143	42 (29∙4)		1∙00	145	99 (68∙3)		1∙00	144	90 (62∙5)	0∙94	1∙00
Unemployed/retired/student	77	30 (39∙0)	1∙66	1∙05, 2∙64	74	27 (36∙5)	2∙96	1∙53, 5∙68	61	46 (75∙4)	0∙81	0∙69, 0∙95	100	31 (31∙0)	0∙78	0∙55, 1∙11	98	24 (24∙5)	0∙83	0∙54, 1∙28	99	65 (65∙7)	0∙96	0∙80, 1∙15	98	65 (66∙3)	1∙06	0∙88, 1∙28
			p-0∙030				p-0∙001				p-0∙008				p-0∙17				p-0∙41				p-0∙67				p-0∙54	
Subsistence farmer/cash cropper	221	106 (47∙9)	2∙05	1∙39, 3∙03	200	36 (18∙0)	1∙46	0∙76, 2∙80	194	139 (71∙7)	0∙77	0∙69, 0∙86	292	23 (7∙9)	0∙20	0∙13, 0∙31	287	59 (20∙6)	0∙70	0∙50, 0∙98	289	183 (63∙3)	0∙93	0∙81, 1∙07	291	176 (60∙5)	0∙97	083, 1∙13
			P<0∙001				p-0∙26				P<0∙001				p<0∙001				p-0∙04				p-0∙0∙30				p-0∙68	
Paid	74	40 (54∙1)	2∙31	1∙51, 3∙52	65	28 (43∙1)	3∙49	1∙83, 6∙64	70	61 (87∙1)	0∙94	0∙84, 1∙04	106	46 (43∙4)	1∙09	0∙81, 1∙47	105	42 (40∙0)	1∙36	0∙96, 1∙92	106	88 (83∙0)	1∙22	1∙06, 1∙40	105	88 (83∙8)	1∙34	1∙15, 1∙56
		p<0∙001	P<0∙001			p<0∙001	P<0∙001			p<0∙001	p-0∙24			p<0∙001	p-0∙56			p-0∙001	p-0∙04			p-0∙003	p-0∙006			p<0∙001	P<0∙001	
**Wealth index (Quintiles)**																												
Lowest	91	46 (50∙6)	1∙00		76	22 (29∙0)	1∙00		79	66 (83∙5)	1∙00		125	27 (21∙6)	1∙00		125	26 (20∙8)	1∙00		125	87 (69∙6)	1∙00		126	83 (65∙9)	1∙00	
Second	83	39 (47∙0)	0∙93	0∙68, 1∙26	80	22 (27∙5)	0∙95	0∙58, 1∙57	76	63 (82∙9)	0∙99	0∙86, 1∙14	118	36 (30∙5)	1∙41	0∙92, 2∙17	114	25 (21∙9)	1∙05	0∙65, 1∙72	116	77 (66∙4)	0∙95	0∙80, 1∙13	116	81 (69∙8)	1∙06	0∙89, 1∙26
			p-0∙64				p-0∙84				p-0∙91				p-0∙12				p-0∙83				p-0∙59				p-0∙51	
Third	81	32 (39∙5)	0∙78	0∙56, 1∙10	79	18 (22∙8)	0∙79	0∙46, 1∙35	78	59 (75∙6)	0∙91	0∙77, 1∙06	122	28 (23∙0)	1∙06	0∙67, 1∙69	120	33 (27∙5)	1∙32	0∙84, 2∙07	121	86 (71∙1)	1∙02	0∙87, 1∙20	121	83 (68∙6)	1∙04	0∙87, 1∙24
			p-0∙15				p-0∙383				p-0∙22				p-0∙80				p-0.22				p-0∙00				p-0∙65	
Fourth	92	35 (38∙0)	0∙75	0∙54, 1∙05	83	17 (20∙5)	0∙71	0∙41, 1∙23	83	64 (77∙1)	0∙92	0∙79, 1∙08	122	27 (22∙1)	1∙02	0∙64, 1∙64	118	36 (30∙5)	1∙47	0∙95, 2∙27	121	84 (69∙4	0∙99	0∙85, 1∙18	121	78 (64∙5)	0∙98	0∙82, 1∙17
			p-0∙09				p-0∙22				p-0∙30				p-0∙92				p-0∙09				p-0∙98				p-0∙82	
Highest	93	35 (37∙6)	0∙74	0∙53, 1∙04	82	18 (22∙0)	0∙76	0∙44, 1∙30	81	61 (75∙3)	0∙90	0∙77, 1∙06	122	31 (25∙4)	1∙18	0∙75, 1∙85	120	36 (30∙0)	1∙44	0∙93, 2∙23	122	85 (69∙7)	1∙00	0∙85, 1∙18	119	80 (67∙2)	1∙02	0∙85, 1∙22
		p-0∙28	p-0∙08			p-∙.67	p-0∙32			p-0∙55	p-0∙20			p-0∙48	p-0∙48			p-0∙28	p-0∙10			p-0∙96	p-0∙99			p-0∙91	p-0∙82	

! Daily tobacco smoking

≠ Consumption of alcohol within last 30 days

¥ Chewed betelnut within last 30 days

# Less fruits/vegetables–consumption of fruits and vegetables <5 days/week

€ Sugar intake > 6 teaspoons of sugar daily or drinking ≥3 soft drinks/week

£ Salt intake–adding salt/maggie stock cubes directly to food daily

β Consumption of fried food (purchased or cooked at home) ≥5days/week.

Pearson’s chi-square or Fisher’s exact where appropriate for prevalence data and p-value. RR = Risk ratio & p-values obtained by binomial regression

Table 3 shows the prevalence and RR of metabolic CVD risk factors by SES. Females were observed to have higher risks for most metabolic CVD risk factors except for hypertension than males (RR 0∙74). They were more likely to have obesity by both WC (RR 5.78) and WHR (RR 1.18), have low HDLc (RR 1.53) and have MetS (RR 4.7) than males. In comparison to Karkar participants, the metabolic CVD risk factors were more common among West Hiri participants, except for central obesity, by WHR (RR 0∙82) and low hdl-c levels (RR 0∙7). West Hiri participants were more likely to have increased WC (RR 2.25), hypertension (RR 2.50), have elevated TC (RR 2.05) and TG (RR 1.43), elevated HbA1c (RR 8.63) and have MetS (RR 2.63) than adults from Karkar Island. Furthermore, adults engaged in paid employment were more likely to be hypertensive (RR 1∙40) but less likely to have obesity by WC (RR 0.49) and WHR (RR 0.79) and have MetS (RR 0∙50) compared to home makers. Conversely, adults in the highest wealth quintile had higher risk of obesity by WC (RR 1∙99) and MetS (RR 1∙84) compared to those in the lowest quintile.

**Table 3:** Univariate analysis of CVD metabolic risk factors by socioeconomic factors (sex, site, education, employment and wealth index).

Socioeconomic characteristics		WC N = 215			WHR N = 442			Hypertension N = 210			Elevated TC N = 105			Elevated TG N = 332			Low HDL-C levels N = 329			Elevated HbA1c N = 24			MetS N = 126	
	N	n (%)	RR (95% CI)	N	n (%)	RR (95% CI)	N	n (%)	RR (95% CI)	N	n (%)	RR (95% CI)	N	n (%)	RR (95% CI)	N	n (%)	RR (95% CI)	N	n (%)	RR (95% CI)	N	n (%)	RR (95% CI)
**Sex**																								
Male	299	27 (9∙0)	1∙00	299	183 (61∙2)	1∙00	294	112 (38∙1)	1∙00	269	43 (16∙0)	1∙00	269	146 (54∙3)	1∙00	270	114 (42∙2)	1∙00	279	8 (2∙9)	1∙00	300	19 (6∙3)	1∙00
Female	360	188 (52∙2)	5∙78 (3∙98, 3∙40)	360	259 (71∙9)	1∙18 (1.05, 1∙31)	346	98 (28∙3)	0∙74 (0.60, 0∙93)	333	62 (18∙6)	1∙16 (0.82, 1∙66)	333	186 (55∙9)	1∙03 (0∙89, 1∙19)	333	215 (64∙6)	1∙53 (1∙30, 1∙80)	340	16 (4∙7)	1∙64 (0∙71, 3∙78)	360	107 (29∙7)	4∙70 (2∙95, 7∙46)
		p<0∙001	p<0∙001		p-0∙003	p-0∙004		p-0∙009	p-0∙009		p-0∙40	p-0∙40		p-0∙70	p-0∙699		p-<0∙001	p-<0∙001		p-0∙297	p-0∙24		p-<0∙001	p-<0∙001
Site																								
Karkar Island	193	38 (19∙7)	1∙00	193	121 (62∙7)	1∙00	199	32 (16∙1)	1∙00	168	19 (11∙3)	1∙00	169	73 (43∙2)	1∙00	169	106 (62∙7)	1∙00	189	2 (1∙1)	1∙00	194	20 (10∙3)	1∙00
West Hiri	251	111 (44∙2)	2∙25 (1∙64, 3∙08)	251	129 (51∙4)	0∙82 (0.70, 0∙96)	251	101 (40∙2)	2∙50 (1.76, 3∙56)	246	57 (23∙2)	2∙05 (1.27, 3∙31)	245	151 (61∙6)	1∙43 (1.17, 1∙74)	246	108 (43∙9)	0∙70 (0∙58, 0∙84)	241	22 (9∙13)	8∙63 (2∙05, 36∙22)	251	68 (27∙1)	2∙63 (1∙66, 4∙17)
			p<0∙001			p-0∙016			p<0∙001			p-0∙003			p<0∙001			p<0∙001			p-0∙003			p<0∙001
Asaro ±	215	66 (30∙7)	1∙56 (1∙10, 2∙21)	215	192 (89∙3)	1∙42 (1.27, 1∙60)	190	77 (40∙5)	2∙52 (1.76, 3∙62)	188	29 (15∙4)	1∙36 (0∙80, 2∙34)	188	108 (57∙5)	1∙33 (1∙08, 1∙64)	188	115 (61∙2)	0∙98 (0∙83, 1∙15)	189	0 (0∙0)	N/A	215	38 (17∙7)	1∙71 (1∙03, 2∙84)
		p<0∙001	p-0∙013		p<0∙001	p<0∙001		p<0∙001	p<0∙001		p-0∙005	p-0∙2604		p-0∙001	p-0∙008		p<0∙001	p-0∙7634		p<0∙001	N/A		p<0∙001	p-0∙04
Education																								
Primary of lower	442	130 (29∙4)	1∙00	442	308 (69∙7)	1∙00	425	126 (29∙7)	1∙00	399	71 (17∙8)	1∙00	400	226 (56∙5)	1∙00	400	225 (56∙3)	1∙00	416	15 (3∙6)	1∙00	442	79 (17∙9)	1∙00
Some/completed Secondary	139	52 (37∙4)	1∙27 (0∙98, 1∙65)	139	80 (57∙6)	0∙83 (0.71, 0∙96)	137	43 (31∙4)	1∙06 (0∙79, 1∙41)	133	26 (19∙6)	1∙10 (0∙73, 1∙65)	132	68 (51∙5)	0∙91 (0∙76, 1∙10)	133	66 (49∙6)	0∙88 (0∙73, 1∙07)	134	6 (4∙5)	1∙24 (0∙49, 3∙14)	140	29 (20∙7)	1∙16 (0∙79, 1∙70)
			p-0∙07			p-0∙02			p-0∙70			p-0∙66			p-0∙33			p-0∙20			p-0∙65			p-0∙45
Vocational/Tertiary±	25	10 (40∙0)	1∙36 (0∙82, 2∙25)	25	17 (68∙0)	0∙98 (0∙74, 1∙29)	25	11 (44∙0)	1∙48 (0.93, 2∙36)	24	2 (8∙3)	0∙47 (0∙12, 1∙79)	24	13 (54∙2)	0∙96 (0∙66, 1∙40)	24	14 (58∙3)	1∙04 (0∙73, 1∙47)	23	0 (0∙0)	N/A	25	4 (16∙0)	0∙90 (0∙36, 2∙25)
		p-0∙13	p-0∙23		p-0∙03	p-0∙86		p-0∙31	p-0∙10		p-0∙45	p-0∙27		p-0∙59	p-0∙83		p-0∙383	p-0∙84		p-0∙69	N/A		p-0∙73	p-0∙81
Employment																								
Home duties	146	89 (61∙0)	1∙00	146	103 (70∙6)	1∙00	142	42 (29∙6)	1∙00	136	33 (24∙3)	1∙00	136	81 (59∙6)	1∙00	136	80 (58∙8)	1∙00	140	9 (6∙4)	1∙00	146	53 (36∙3)	1∙00
Unemployed/retire/student	98	16 (16∙3)	0∙27 (0∙17, 0∙43)	98	52 (53∙1)	0∙75 (0∙61, 0∙93)	98	23 (23∙5)	0∙79 (0∙51, 1∙23)	87	15 (17∙2)	0∙71 (0∙41, 1∙23)	88	50 (56∙8)	0∙95 (0∙76, 1∙20)	88	39 (44∙3)	0∙75 (0∙57, 0∙99)	90	3 (3∙3)	0∙52 (0∙14, 1∙86)	98	8 (8∙2)	0∙22 (0∙11, 0∙45)
			p<0∙001			p-0∙009			p-0.30			p-0∙22			p-0∙69			p-0∙04			p-0∙31			p<0∙001
Subsistence farmer/cropper	288	72 (25∙0)	0∙41 (0∙32, 0∙52)	288	208 (72∙2)	1∙02 (0∙90, 1∙16)	273	92 (33∙7)	1∙14 (0∙84, 1∙54)	263	35 (13∙3)	0∙55 (0∙36, 0∙84)	263	137 (52∙6)	0∙87 (0∙73, 1∙05)	263	153 (58∙2)	0∙99 (0∙83, 1∙18)	273	2 (0∙7)	0∙11 (0∙02, 0∙52)	289	44 (15∙2)	0∙42 (0∙30, 0∙59)
			p-0∙001			p-0∙72			p-0∙40			p-0∙006			p-0∙15			p-0∙90			p-0∙005			p-0∙001
Paid	104	31 (29∙8)	0∙49 (0∙35, 0∙67)	104	58 (55∙8)	0∙79 (0∙65, 0∙97)	104	43 (41∙4)	1∙40 (0∙99, 1∙97)	100	21 (21∙0)	0∙87 (0∙53, 1∙40)	99	55 (55∙6)	0∙93 (0∙75, 1∙17)	100	48 (48∙0)	0∙82 (0∙64, 1∙05)	100	8 (8∙0)	1∙24 (0∙50, 3∙11)	104	19 (18∙3)	0∙50 (0∙32, 0∙80)
		p<0∙001	p-0∙001		p<0∙001	p-0∙02		p-0∙04	p-0∙06		p-0∙04	p-0∙56		p-0∙54	p-0∙54		p-0∙05	p-0∙11		p<0∙001	p-0∙64		p<0∙001	p-0∙003
Wealth index (Quintiles) Overall																								
Lowest	123	27 (22∙0)	1∙00	123	86 (69∙2)	1∙00	122	40 (32∙8)	1∙00	110	20 (18∙2)	1∙00	110	53 (48∙2)	1∙00	110	55 (50∙0)	1∙00	114	2 (1∙8)	1∙00	124	17 (13∙7)	1∙00
Second	117	34 (29∙1)	1∙32 (0∙85, 2∙05)	117	71 (60∙7)	0∙87 (0∙72, 1∙05)	113	37 (32∙7)	0∙99 (0∙69, 1∙44)	106	18 (17∙0)	0.93 (0∙52, 1∙67)	107	59 (55∙1)	1∙14 (0∙88, 1∙48)	107	55 (51∙4)	1∙03 (0∙79, 1∙34)	108	6 (5∙6)	3.17 (0∙65, 15∙35)	117	21 (18∙0)	1∙31 (0∙73, 2∙36)
			p-0∙21			p-0∙14			p-0∙99			p-0∙82			p-0∙31			p-0∙84			p-0∙15			p-0∙37
Third	120	39 (32∙5)	1∙48 (0∙97, 2∙26)	120	75 (62∙5)	0∙89 (0∙75, 1∙07)	116	37 (31∙9)	0∙97 (0∙67, 1∙41)	114	21 (18∙4)	1∙01 (0∙58, 1∙76)	114	63 (55∙3)	1∙15 (0∙89, 1∙48)	114	59 (51∙8)	1∙04 (0∙80, 1∙34)	116	5 (4∙3)	2∙46 (0∙49, 12∙41)	120	24 (20∙0)	1∙46 (0∙83, 2∙58)
			p-0∙07			p-0∙22			p-0∙88			p-0∙96			p-0∙29			p-0∙79			p-0∙28			p-0∙19
Fourth	120	45 (37∙5)	1∙71 (1∙14, 2∙56)	120	88 (73∙3)	1∙05 (0∙90, 1∙23)	112	34 (30∙4)	0∙93 (0∙63, 1∙35)	111	16 (14∙4)	0∙79 (0∙43, 1∙45)	111	66 (59∙5)	1∙23 (0∙96, 1∙58)	111	66 (59∙5)	1∙19 (0∙93, 1∙51)	110	1 (0∙9)	0.52 (0∙05, 5∙63)	120	25 (20∙8)	1∙52 (0∙87, 2∙67)
			p-0∙01			p-0∙56			p-0∙69			p-0∙82			p-0∙10			p-0∙16			p-0∙59			p-0∙15
Highest	119	52 (43∙7)	1∙99 (1∙35, 2∙94)	119	81 (68∙1)	0∙97 (0∙82, 1∙15)	119	43 (36∙1)	1∙10 (0∙78, 1∙56)	106	18 (17∙0)	0∙93 (0∙52, 1∙67)	105	57 (54∙3)	1∙13 (0∙87, 1∙46)	106	63 (59∙4)	1∙19 (0∙93, 1∙52)	115	7 (6∙1)	3.47 (0∙74, 16∙35)	119	30 (25∙2)	1∙84 (1∙07, 3∙15)
		p-0∙005	p-0∙001		p-0∙20	p-0∙76		p-0∙92	p-0∙59		p-0∙939	p-0∙82		p-0∙57	p-0∙37		p-0∙43	p-0∙17		p-0∙14	p-0∙12		p-0∙24	p-0∙03

WC = Waist circumference ≥94 cm (males) & ≥80cm (females); WHR = waist-to-hip ratio ≥∙90 (males) & ≥∙85; Hypertension ≥130/85mmHg; TC = total cholesterol >6∙2mm/L; TG = total triglyceride ≥1∙7 mmol/L; HDL-c = high density lipoprotein cholesterol <1mmol/L (males) & <1∙3mmol/L; HbA1c = haemoglobin A1c ≥6∙5%; MetS = metabolic syndrome i.e. obesity + any two of the other risk factors; Pearson’s chi-square or Fisher’s exact where appropriate for prevalence data and p-value. RR = Risk ratio & p-values obtained by binomial regression. ± No cases of diabetes in Asaro, vocational/tertiary education, therefore analysis excluded them N/A = Not applicable

The multivariate analysis showed West Hiri and Asaro adults, respectively had higher risk for low fruit/vegetable consumption (RR 102.9 & RR 13.7), high alcohol use (RR 7.93 & RR 3.20), high sugar (RR 4.63 & RR 2.49), salt (RR 3.50 & RR 3.72) and fried food intake (RR 52.6 & RR 56.2), obesity (WC) (RR 1.67 & RR 1.70), hypertension (RR 2.93 & RR 2.60), high cholesterol (RR 2.22 & RR 1.79), triglyceride (RR 1.49 & RR 1.34), and MetS (RR 2.04 & RR 1.88) compared to Karkar adults. (Table 4) Multivariate analysis showed adults with higher education had less risk of having elevated HbA1c levels (RR 3.04e-08) but 1∙12 times the risk of eating fried food than those with lower than primary education. Adults in paid employment had 43% reduced risk of eating less fruits and vegetables compared to home makers. Adults in the highest wealth quintiles had 1∙55, 1∙98 and 1∙70 times the risk of having high sugar intake, obesity (WC) and MetS, respectively, compared to those in lowest quintile.

**Table 4:** Multivariate analysis of CVD behavioral and metabolic risk factors by socioeconomic factors adjusted for sex and age and site.

Socioeconomic characteristics	Tobacco smoking!	Alcohol use ≠	Betel nut chewing ¥	Less fruit/vegetables intake #	High sugar intake €	Salt intake £	Fried food intake β	WC	WHR	High BP	Elevated TC	Elevated TG	Low HDL-C levels	Elevated HbA1c	MetS
	N = 390	N = 358	N = 350	N = 546	N = 537	N = 542	N = 542	N = 538	N = 538	N = 521	N = 493	N = 493	N = 494	N = 509	N = 539
	RR (95% CI)	RR (95% CI)	RR (95% CI)	RR (95% CI)	RR (95% CI)	RR (95% CI)	RR (95% CI)	RR (95% CI)	RR (95% CI)	RR (95% CI)	RR (95% CI)	RR (95% CI)	RR (95% CI)	RR (95% CI)	RR (95% CI)
**Sex**															
Male	1∙00	1∙00	1∙00	1∙00	1∙00	1∙00	1∙00	1∙00	1∙00	1∙00	1∙00	1∙00	1∙00	1∙00	1∙00
Female	0∙37 (0∙28, 0∙49)	0∙18 (0∙09, 0∙35)	0∙97 (0∙86, 1∙10)	0∙68 (0∙47, 0∙99)	0∙51 (0∙35, 0∙75)	1∙00 (0∙90, 1∙10)	1∙01 (0∙96, 1∙07)	6∙28 (4∙08, 9∙66)	1∙11 (0∙97, 1∙26)	0∙74 (0∙54, 1∙01)	0∙81 (0∙50, 1∙32)	0∙91 (0∙74, 1∙11)	1∙64 (1∙36, 1∙98)	1∙02 (0∙28, 3∙71)	4∙64 (2∙71, 7∙93)
p-value	p<0∙001	p<0∙001	p-0∙62	p-0∙05	p-0∙001	p-0∙93	p-0∙64	p<0∙001	p-0∙12	p-0∙06	p-0∙40	p-0∙33	p<0∙001	p-0∙98	p<0∙001
**Site**															
Karkar Island	1∙00	1∙00	1∙00	1∙00	1∙00	1∙00	1∙00	1∙00	1∙00	1∙00	1∙00	1∙00	1∙00	1∙00	1∙00
West Hiri	0∙77 (0∙55, 1∙08)	7∙93 (4∙13, 15∙24)	1∙05 (0∙93, 1∙19)	102∙9 (13∙67, 774∙ 50)	4∙63 (2∙74, 7∙84)	3∙50 (2∙65, 4∙63)	52∙56 (16∙90, 163∙52)	1∙67 (1∙21, 2∙31)	0∙722 (0∙59, 0∙87)	2∙93 (1∙89, 4∙56)	2∙22 (1∙20, 4∙10)	1∙49 (1∙17, 1∙91)	0∙63 (0∙50, 0∙79)	5∙42 (1∙36, 21∙56)	2∙04 (1∙25, 3∙32)
pvalue	p-0∙13	p<0∙001	p-0∙40	p<0∙001	p<0∙001	p<0∙001	p<0∙001	p-0∙002	p-0∙001	p<0∙001	p-0∙01	p-0∙001	p<0∙001	p-0∙017	p-0∙004
Asaro	0∙73 (0∙57, 0∙93)	3∙20 (1∙53, 6∙67)	0∙68 (0∙57, 0∙80)	13∙71 (1∙85, 101∙52)	2∙49 (1.46, 4∙25)	3∙72 (2∙84, 4∙86)	56∙21 (18∙37, 171∙95)	1∙70 (1∙21, 3∙38)	1∙48 (1∙28, 1∙70)	2∙60 (1∙71, 3∙95)	1∙79 (0∙97, 3∙29)	1∙34 (1∙06, 1∙70)	1∙02 (0∙85, 1∙22)	4∙18e-07 (4∙45e-08, 3∙92e-06)	1∙88 (1∙12, 3∙16)
pvalue	p-0∙011	p-0∙002	P<0∙001	p-0∙010	p-0∙001	p<0∙001	p<0∙001	p-0∙002	p<0∙001	p<0∙001	p-0∙06	p-0∙013	p-0∙81	p<0∙001	p-0∙016
**Education**															
Primary/lower	1∙00	1∙00	1∙00	1∙00	1∙00	1∙00	1∙00	1∙00	1∙00	1∙00	1∙00	1∙00	1∙00	1∙00	1∙00
Some/completed secondary	0∙84 (0∙64, 1∙10)	0∙81 (0∙53, 1∙24)	1∙04 (0∙93, 1∙15)	0∙91 (0∙70, 1∙18)	0∙90 (0∙64, 1∙27)	1∙10 (0∙99, 1∙23)	1∙05 (0∙97, 1∙14)	1∙22 (0∙94, 1∙58)	0∙97 (0∙81, 1∙16)	0∙96 (0∙68, 1∙35)	0∙81 (0∙49, 1∙31)	0∙82 (0∙66, 1∙03)	1∙05 (0∙85, 1∙30)	0∙66 (0∙23, 1∙87)	1∙08 (0∙72, 1∙62)
pvalue	p-0∙21	p-0∙33	p-0∙51	p-0∙47	p-0∙54	p-0∙09	p-0∙26	p-0∙14	p-0∙73	p-0∙81	p-0∙38	p-0∙09	p-0∙64	p-0∙43	p-0∙71
Vocational/tertiary	0∙58 (0∙30, 1∙10)	0∙99 (0∙62, 1∙57)	0∙88 (0∙67, 1∙14)	1∙01 (0∙66, 1∙56)	0∙70 (0∙35, 1∙42)	1∙18 (0∙99, 1∙42)	1∙12 (1∙04, 1∙21)	1∙31 (0∙78, 2∙21)	1∙17 (0∙84, 1∙62)	1∙17 (0∙72, 1∙89)	0∙19 (0∙93, 1∙19)	0∙79 (0∙51, 1∙23)	1∙09 (0∙68, 1∙74)	3∙04e-08 (1∙37e-08, 6∙73e-08)	0∙66 (0∙23, 1∙94)
pvalue	p-0∙09	p-0∙96	p-0∙33	p-0∙95	p-0∙32	p-0∙07	p-0∙002	p-0∙30	p-0∙36	p-0∙53	p-0∙08	p-0∙30	p-0∙73	p<0∙001	p-0∙45
**Employment**															
Home duties	1∙00	1∙00	1∙00	1∙00	1∙00	1∙00	1∙00	1∙00	1∙00	1∙00	1∙00	1∙00	1∙00	1∙00	1∙00
Unemployed/retired/student	0∙81 (0∙47, 1∙38)	0∙71 (0∙28, 1∙75)	0∙79 (0∙64, 0∙98)	0∙61 (0∙38, 0∙98)	0∙53 (0∙64, 1∙66)	0∙93 (0∙79, 1∙10)	1∙08 (0∙96, 1∙21)	0∙71 (0∙39, 1∙28)	0∙78 (0∙59, 1∙03)	0∙77 (0∙43, 1∙38)	0∙95 (0∙51, 1∙66)	0∙99 (0∙71, 1∙40)	0∙98 (0∙69, 1∙39)	2∙02e-07 (4∙09e-08, 9∙99e-07)	0∙54 (0∙18, 1∙59)
pvalue	p-0∙43	p-0∙452	p-0∙03	p-0∙04	p-0∙91	p-0∙40	p-0∙23	p-0∙26	p-0∙09	p-0∙39	p-0∙91	p-0∙97	p-0∙91	p<0∙001	0∙27
Subsistence farmer/cropper	1.08 (0∙66, 1∙77)	0∙83 (0∙34, 2∙00)	0∙91 (0∙81, 1∙03)	0∙61 (0∙37, 1∙02)	0∙75 (0∙44, 1∙27)	0∙97 (0∙82, 1∙15)	1∙05 (0∙97, 1∙13)	0∙85 (0∙62, 1∙17)	0∙78 (0∙66, 0∙93)	1∙03 (0∙67, 1∙59)	0∙59 (0∙31, 1∙11)	0∙87 (0∙67, 1∙12)	0∙98 (0∙80, 1∙20)	0∙24 (0∙03, 2∙09)	0∙79 (0∙49, 1∙27)
pvalue	p-0∙76	p-0∙67	p-0∙15	p-0∙06	p-0∙29	p-0∙73	p-0∙27	p-0∙32	p-0∙004	p-0∙90	p-0∙10	p-0∙28	p-0∙82	p-0∙20	p-0∙33
Paid	1∙39 (0.85, 2∙27)	0∙62 (0∙25, 1∙51)	0∙91 (0∙78, 1∙05)	0∙57 (0∙38, 0∙86)	0∙68 (0∙41, 1∙14)	0∙92 (0∙78, 1∙08)	1∙01 (0∙91, 1∙12)	1∙16 (0∙80, 1∙70)	0∙87 (0∙67, 1∙14)	0∙88 (0∙56, 1∙38)	0∙90 (0∙45, 1∙79)	0∙83 (0∙61, 1∙14)	1∙19 (0∙87, 1∙63)	1∙57 (0∙46, 5∙34)	1∙18 (0∙68, 2∙07)
pvalue	p-0∙19	p-0∙292	p-0∙19	p-0∙008	p-0∙14	p-0∙30	p-0∙88	p-0∙43	p-0∙33	p-0∙58	p-0∙76	p-0∙26	p-0∙28	p-0∙47	p-0∙56
**Wealth index (quintiles)**															
Lowest	1∙00	1∙00	1∙00	1∙00	1∙00	1∙00	1∙00	1∙00	1∙00	1∙00	1∙00	1∙00	1∙00	1∙00	1∙00
Second lowest	0∙93 (0∙71, 1∙23)	1∙11 (0∙70, 1∙75)	0∙98 (0∙86, 1∙12)	1∙57 (1∙09, 2∙24)	1∙03 (0∙64, 1∙66)	0∙92 (0∙79, 1∙08)	1∙09 (1∙00, 1∙18)	1∙19 (0∙77, 1∙85)	0∙87 (0∙73, 1∙03)	1∙02 (0∙69, 1∙50)	0∙92 (0∙51, 1∙66)	1∙05 (0∙81, 1∙36)	1∙01 (0∙78, 1∙31)	1∙50 (0∙34, 6∙55)	1∙10 (0∙61, 2∙00)
pvalue	p-0∙62	p-0∙66	p-0∙77	p-0∙014	p-0∙91	p-0∙31	p-0∙05	p-0∙43	p-0∙11	p-0∙92	p-0∙78	p-0∙72	p-0∙94	p-0∙59	p-0∙75
Third	0∙88 (0∙65, 1∙18)	0∙89 (0∙53, 1∙48)	0∙93 (0∙81, 1∙03)	1∙12 (0∙74, 1∙59)	1∙35 (0∙87, 2∙07)	1∙02 (0∙89, 1∙17)	1∙05 (0∙97, 1∙14)	1∙48 (1∙02, 2∙16)	0∙92 (0∙78, 1∙09)	0∙94 (0∙63, 1∙39)	1∙01 (0∙58, 1∙77)	1∙08 (0∙85, 1∙39)	1∙05 (0∙81, 1∙35)	1∙75 (0∙39, 7∙89)	1∙32 (0∙78, 2∙23)
pvalue	p-0∙40	p-0∙65	p-0∙30	p-0∙59	p-0∙18	p-0∙75	p-0∙22	p-0∙04	p-0∙34	p-0∙76	p-0∙96	p-0∙53	p-0∙71	p-0∙47	p-0∙31
Fourth	0∙79 (0∙57, 1∙08)	0∙77 (0∙46, 1∙27)	0∙90 (0∙77, 1∙04)	1∙07 (0∙70, 1∙62)	1∙37 (0∙88, 2∙13)	1∙03 (0∙89, 1∙18)	0∙99 (0∙90, 1∙09)	1∙60 (1∙08, 2∙37)	1∙05 (0∙90, 1∙23)	0∙93 (0∙63, 1∙37)	0∙89 (0∙48, 1∙63)	1∙18 (0∙92, 1∙51)	1∙19 (0∙93, 1∙51)	0∙42 (0∙04, 4∙60)	1∙35 (0∙77, 2∙36)
pvalue	p-0∙14	p-0∙30	p-0∙161	p-0∙76	p-0∙16	p-0∙73	p-0∙77	p-0∙02	p-0∙52	p-0∙71	p-0∙71	p-0∙19	p-0∙17	p-0∙48	p-0∙30
Highest	0∙79 (0∙56, 1∙10)	0∙73 (0∙40, 1∙31)	0∙88 (0∙74, 1∙04)	1∙32 (0∙88, 1∙99)	1∙55 (1∙00, 2∙41)	0∙94 (0∙82, 1∙08)	1∙03 (0∙93, 1∙13)	1∙98 (1∙36, 2∙88)	1∙00 (0∙84, 1∙18)	1∙21 (0∙83, 1∙76)	0∙98 (0∙53, 1∙81)	1∙13 (0∙86, 1∙47)	1∙17 (0∙91, 1∙49)	1∙53 (0∙30, 7∙96)	1∙70 (1∙00, 2∙88)
pvalue	p-0∙16	p-0∙29	p-0∙126	p-0∙18	p-0∙049	p-0∙38	p-0∙60	P<0∙001	p-0∙99	p-0∙33	p-0∙94	p-0∙39	0∙23	p-0∙61	p-0∙048

WC = Waist circumference ≥94 cm (males) & ≥80cm (females); WHR = waist-to-hip ratio ≥∙90 (males) & ≥∙85; Hypertension ≥130/85mmHg; TC = total cholesterol >6·2mm/L; TG = total triglyceride ≥1·7mmol/L; HDL-c = high density lipoprotein cholesterol <1mmol/L (males) & <1·3mmol/L; HbA1c = haemoglobin A1c ≥6·5%; MetS = metabolic syndrome i.e. obesity + any two of the other risk factors; RR = Risk ratio & p-values obtained by modified Poisson regression with a robust error variance.

! Daily tobacco smoking

≠ Consumption of alcohol within last 30 days

¥ Chewed betelnut within last 30 days

# Less fruits/vegetables–consumption of fruits and vegetables <5 days/week

€ Sugar intake > 6 teaspoons of sugar daily or drinking ≥3 soft drinks/week

£ Salt intake–adding salt/maggie stock cubes directly to food daily

β Consumption of fried food (purchased or cooked at home) ≥5days/week

## Discussion

We have previously reported on the prevalence of NCD risk factors in three different regions of PNG [20]. We observed high prevalence of low fruit and vegetable intake, high intake of sugar, salt and fried food and alcohol, obesity (WC), hypertension and low HDL-c levels among those with higher education compared to adults in the lowest education group. However, tobacco smoking, central obesity (WHR) and high triglyceride levels were more prevalent among adults with lower education. Furthermore, among those in paid employment, we observed higher prevalence of tobacco smoking, alcohol use, less fruits and vegetables intake, high intake of sugar, salt and fried food, hypertension and elevated HbA1c levels. In addition, we found high sugar intake, obesity (WC), hypertension, elevated HbA1c levels and MetS was more prevalent among the highest wealth quintile group compared to those in the lowest quintile group. However, high levels of tobacco smoking, alcohol use and betelnut chewing and high TC were more prevalent among adults in the lowest wealth quintile group.

The patterning of CVD risk factors in our study supports the notion that risk factors and disease risk are impacted differently according to the influences of socioeconomic transitions experienced by different individuals and their communities. A recent review of the association between CVD and SES in LMICs reported that people in low SES groups generally had higher prevalence of alcohol, tobacco smoking and consumed less vegetables and fruits while adults of high SES groups consume more processed food and were less physically active.[11] Furthermore, reviews of studies which were conducted in other LMICs have also shown large variation in these associations where the direction of the associations differed from one country to another and differed for the different CVD risk factors.[11, 12, 45]

A study in India which collected data from participants aged 26–32 between 1998 to 2002, observed higher prevalence of CVD risk factors such as obesity and hypertension among those with higher SES[46], but the pattern did not hold for tobacco smoking. Similar findings were observed in a more recent study which looked at the socio-demographic patterning of CVD risk factors among rural populations from major cities in India.[47] This variability in the CVD risk factors is also observed in our findings across the three study samples. For example, adults with higher educational attainment were observed to have high salt and fried food intake, consume less fruits and vegetables, but were less likely to smoke tobacco, have high TC and HbA1c levels.

Some of our findings differ to those from other LMICs such as that observed in a study among adults in Aleppo, Syria in 2006.[48] The Aleppo study observed an inverse association between education and CVD risk factors. Their results showed diabetes, obesity, hypertension and high cholesterol levels were highly prevalent among people with low education levels. An inverse association was also observed between employment status and CVD risk factors, with a higher prevalence of hypertension and diabetes among those with no employment compared to the employed.[48] In contrast, our results showed adults with paid employment had a higher prevalence of tobacco smoking, alcohol use, consumed less fruits and vegetables, high sugar, salt and fried food intake, obesity, hypertension, HbA1c and MetS compared to adults with home duties.

An article published in 2012 which looked at the association between CVD risk factors and education, occupational and SES in an Asian Indian population, reported these risk factors to be highly prevalent among adults with low SES.[49] The study observed a positive association between low SES and truncal obesity, high triglyceride and low HDL-c levels, tobacco smoking and low physical activity. These findings were in contrast to earlier studies from India that reported people in lower SES groups had a lower CVD risks.[50]

Urbanization in PNG appears to be accelerating the CVD risk in rural areas to levels comparable to longer-term urban or peri-urban populations, such as that seen in other LMICs.[51, 52] As economic development progresses, the SES gradient tends to become more regressive.(9, 12) This is clearly illustrated by Kerr GD et al and Allen L et al.[8, 11] These two articles reviewed studies which looked at the association of SES and NCD and risks factors which showed that at advanced stages of socioeconomic transition (HIC), there are clear SES gradients of NCDs and risk factors [8] while in LMIC, countries are at different stages of these emerging, hence mix trends are observed.[11] It is apparent that different stages of socioeconomic transition produce different distributions of risk factors across different SES groups, thus adds to the understanding of which groups change which behaviors at these stages of transition. In the current study, we observed mixed findings for the association between SES and CVD risk factors.

A recent study by Gouda et al (submitted and will be published in due course) of verbal autopsy studies in PNG, from 1970–2001 and 2009–2014, showed that an epidemiological transition in mortality is underway in PNG. Deaths from NCDs, particularly diabetes, stroke and ischaemic heart disease, are on the rise, more so in peri-urban West Hiri and Asaro than Karkar Island sites. This mortality data is evidence of the existing and high prevalence of NCD risk factors among populations who have had a greater and increasing exposure to modernization and earning an income through employment or cash crops as observed in our study.(20)

### Strengths and limitations

The strengths of this study include our use of robust objective measures, standard methods and instruments to collect data as well as blood sample analysis. The limitations include the cross-sectional design, limiting our ability to draw causal inferences, and therefore, only providing a snapshot in a rapidly-changing society. In addition, the target sample of 900 adults was not achieved. It proved difficult to have study participants fasting for the blood sample collection, therefore non-fasting blood samples were used for the analysis. The behavioral risk factors were self-reported using standardized questions. Finally, we created a wealth index from household data, an approach that is commonly used, but was challenging to construct and interpret, and the possibility of misclassification across the wealth quintiles could be a threat to validity.

## Conclusion

The findings from this study highlight variability in CVD risk factor prevalence that is not fully explained by SES, therefore, further studies in such settings would enable a better understanding of these associations. Our findings suggest that all community members are at risk of CVD whether they are part of high or low SES groups. Such changes should be of major concern for PNG policy makers as it suggests the rise in CVD is not confined to urban areas. To conclude, this study provides baseline from which trends can be observed. If the adverse health impacts from consumption of alcohol, tobacco and processed foods are to be mitigated equitably, whole of population strategies, such as those identified by WHO as “Best Buys”, need to be implemented throughout PNG, but with consideration of adapting them where needed to fit diverse local contexts.

## Supporting information

S1 Dataset Minimal anonymized data of 671 study participants. (XLSX)
