# Correction: Socio-economic status and behavioural and cardiovascular risk factors in Papua New Guinea: A cross-sectional survey

**DOI:** 10.1371/journal.pone.0212894

**Published:** 2019-02-20

**Authors:** Patricia Rarau, Justin Pulford, Hebe Gouda, Suparat Phuanukoonnon, Chris Bullen, Robert Scragg, Bang Nguyen Pham, Barbara McPake, Brian Oldenburg

The fourth author's name is spelled incorrectly. The correct name is: Suparat Phuanukoonnon. The correct citation is: Rarau P, Pulford J, Gouda H, Phuanukoonnon S, Bullen C, Scragg R, et al. (2019) Socio-economic status and behavioural and cardiovascular risk factors in Papua New Guinea: A cross-sectional survey. PLoS ONE 14(1): e0211068. https://doi.org/10.1371/journal.pone.0211068

There is an error in the current address for author Suparat Phuanukoonnon. The correct current address is: Department of Social and Environmental Medicine, Faculty of Tropical Medicine, Mahidol University, Ratchadewee, Bangkok, Thailand.
